# Supplementary material for: The outcomes of SGLT-2 inhibitor utilization in diabetic kidney transplant recipients
Source: Nat Commun. 2024 Nov 20;15:10043. doi: 10.1038/s41467-024-54171-8 (PMC11579355; doi:10.1038/s41467-024-54171-8)
Supplement: Supplementary file 1 — Supplementary Information [file 41467_2024_54171_MOESM1_ESM.pdf]

**Supplementary Appendix to "The Outcomes of SGLT-2 Inhibitor Utilization in Diabetic Kidney Transplant Recipients"**

TriNetX is the global federated health research network providing access to electronic medical records (diagnoses, procedures, medications, laboratory values, genomic information) across large healthcare organizations (HCOs). It provides a diverse range of data, including patient demographics, medical diagnoses (coded using ICD-10-CM), various medical procedures (recorded with ICD-10 Procedure Coding System, Current Procedural Terminology codes, or Systematized Nomenclature of Medicine - Clinical Terms), prescribed drugs (categorized by the Anatomical Therapeutic Chemical Classification system or RxNorm), laboratory tests (classified by Logical Observation Identifiers Names and Codes), genetic data (notated according to the Human Genome Variation Society standards), and detailed records of healthcare service use. This report was run on the set of HCOs grouped into a network called Research. This network included 83 HCO(s). The analysis process includes two main steps:
1) Defining the cohorts through query criteria;
2) Setting up and running the analysis.

According to the detailed description of the database provided and can be found at the website: https://trinetx.com/trust-security/, TriNetX is recognized as an ISO/IEC 27001:2013 certified entity, demonstrating its commitment to maintaining a robust Information Security Management System (ISMS). This international standard outlines the criteria for the creation, implementation, operation, surveillance, assessment, preservation, and enhancement of a documented ISMS, tailoring it to the specific risks faced by the organization. Emphasizing a risk-based methodology, ISO/IEC 27001:2013 advocates for suitable and balanced security measures that safeguard information assets, thereby instilling confidence in TriNetX's stakeholders, including its members and clients. The ISMS of TriNetX underpins the architecture and functionalities required to handle the data assets, personnel, operations, and technology that form the backbone of the TriNetX platform. Further information regarding TriNetX's ISO/IEC 27001:2013 Certification can be found in the Schellman ISO Certificate Directory (https://www.schellman.com/certificate-directory).

TriNetX has established systems and protocols that align with the European Union's (EU) criteria for the extraterritorial transfer of data from the EU to its platform, emphasizing the safeguarding and enhancement of the confidentiality, integrity, availability, and resilience of systems and services that process Personal Data. To guarantee that information is adequately de-identified in accordance with the Health Information Portability and Accountability Act (HIPAA) Privacy Rule, TriNetX employs the Expert Determination method. For this purpose, the services of Bradley Malin, Ph.D., are utilized. Furthermore, TriNetX adheres to the standards set by the Brazilian General Data Protection Law (LGPD), demonstrating its commitment to transparency by entering into Data Protection

Addendums with Brazilian healthcare organizations. According to the agreements between HCOs and TriNetX, all data provided to TriNetX will undergo pseudonymization and/or de-identification by the HCOs. TriNetX also offers assistance to HCOs in establishing pseudonymization processes if they are not already in place. Additionally, expert determination is used by TriNetX to ensure that data made available to end-users is sufficiently de-identified. To manage data consistently, TriNetX has formed a Data Trust team, which includes employees from its legal, privacy, compliance, and security departments. This team is tasked with evaluating data throughout its lifecycle, ensuring responsible and accountable data management. This information is based on the source document from TriNetX's official website (https://trinetx.com/trust-privacy/).

**Cohorts definition**

Query Criteria for Cohort 1 (query name: SGLT-2i users):

This query was run on the network Research with 83 HCO(s) queried and 83 HCO(s) responded. A total of 57 provider(s) responded with patients.

Query Criteria for Cohort 2 (query name: SGLT-2i non-users):

This query was run on the network Research with 83 HCO(s) queried and 83 HCO(s) responded. A total of 76 provider(s) responded with patients.

The specific codes used to identify diabetic kidney transplant recipients are outlined in **Supplemental Table S7**, while the codes utilized for querying SGLT-2i use, and the criteria for exclusions are detailed in **Supplemental Table S8**.

**Analysis Setup**

This analysis included outcomes that occurred in the time window that started 90 days after the first occurrence of the index event and ended 1825 days after the first occurrence of the index event. The index event only includes events that occurred up to 20 years ago. Patients whose index event occurred 20 years or more ago are excluded. In this analysis, 0 patients in Cohort 1 and 0 patients in Cohort 2 were excluded because they met the index event more than 20 years ago. Index events for the Compare Outcomes analysis were derived from the cohort definitions. Index events were defined separately for each cohort and were based on the criteria used in the original cohort definition.

The Survival analyses support the “exclude patients with outcomes prior to the window” setting. This option can exclude patients from the analysis if they are not at risk for an outcome (e.g., if the outcome is a chronic disease). When "exclude patients with the outcome prior to the time window" is not checked, all patients in the cohort are included in the analysis, regardless of whether they had the outcome prior to the time window. When "exclude patients with the outcome prior to the time window" is checked, patients are excluded from the analysis if their record includes the outcome

prior to the beginning of the time window. This selection will exclude all patients with the outcome prior to the index event. If the start of the time window for the analysis falls some days after the index event, patients will also be excluded if they have the outcome between the index event and the start of the time window.

The Kaplan-Meier Analysis estimates probability of the outcome at a respective time interval (daily time interval is used in this analysis). In order to account for the patients who exited the cohort during the analysis period, and therefore should not be included in the analysis, censoring is applied. In this analysis, patients are removed from the analysis (censored) after the last fact in their record. The output summary includes: Patients in each Cohort (count of patients meeting query criteria); Patients with Outcome (of the patients in the cohort, count of patients that had the outcome in the time window); Median Survival (the number of days when the survival drops below 50%; the "-" indicates that survival does not drop below 50% during the time window); and Survival Probability at End of Time Window (the % survival at the end of the time window). In addition, Log-Rank test, Hazard Ratio and test for Proportionality.

**Supplemental Table S9** details the criteria for each outcome, specifying that for definitions encompassing multiple terms, the presence of at least one term is required for a match.

For a balanced comparison between the study groups, we incorporated a variety of demographic factors such as age, sex, and ethnic and racial backgrounds. Moreover, we scrutinized various comorbidities and concurrent medication use. We conducted propensity score matching based on 45 characteristics, including demographic factors such as age at Index, race (White), ethnicity (not Hispanic or Latino), and sex (male). Our analysis also encompassed a range of comorbidities, identified through ICD-10-CM codes. These included primary hypertension (I10), dyslipidemia (E78), heart failure (I50), liver diseases (K70-K77), chronic lower respiratory diseases (J40-J4A), systemic connective tissue disorders (M30-M36), neoplasms (C00-D49), overweight and obesity (E66), T2DM-related complications like nephropathy, ophthalmopathy, and neuropathy (E11.2-E11.4), nephrotic syndrome (N04), cystic kidney disease (Q61), and smoking (Z72.0). Medication use was classified according to the Anatomical Therapeutic Chemical Classification System, covering Corticosteroids (ATC:H02), Insulin (ATC:A10A), Biguanides (ATC:A10BA), GLP-1 agonists (ATC:A10BJ), DPP-4 inhibitors (ATC:A10BH), Sulfonylureas (ATC:A10BB), Thiazolidinediones (ATC:A10BG), HMG-CoA reductase inhibitors (ATC:C10AA), ACE inhibitors/ARBs (ATC:C09), Beta-blockers (ATC:C07), Calcium channel blockers (ATC:C08), and Diuretics (ATC:C03). Additionally, we accounted for potential confounding factors by analyzing physical examination findings and laboratory tests, which included systolic and diastolic blood pressure, body mass index (BMI), serum sodium and potassium levels, estimated glomerular filtration rate (eGFR), triglyceride and cholesterol levels, hemoglobin A1c (HbA1c), alanine aminotransferase (ALT), aspartate aminotransferase (AST), total bilirubin, and brain-type natriuretic peptide (BNP). These conditions were verified through diagnoses in the electronic health records of patients, spanning from a year to a day before the index date. The specific diagnosis and medication

codes utilized in our study are summarized in **Supplemental Table S10**.

**Methods for New-User Analysis**

In this analysis, we utilized the TriNetX system to classify kidney transplant recipients into two distinct groups based on their SGLT-2i medication usage prior to the transplant event:

- New SGLT-2i users were defined as patients who had no recorded SGLT-2i medication codes prior to the kidney transplant.
- Long-term SGLT-2i users were defined as patients who had documented SGLT-2i medication codes before the transplant event.

This classification was conducted to allow a new-user design subgroup analysis, enhancing the clarity and precision of the study results by distinguishing between the effects of initiating SGLT-2i after transplant versus continued use from pre-transplant periods. The outcomes of interest, including all-cause mortality, major adverse cardiovascular events (MACE), and major adverse kidney events (MAKE), were assessed with hazard ratios adjusted for age, sex, and race due to their potential interactions with kidney disease outcomes. The adjusted hazard ratios and 95% confidence intervals were calculated using Cox proportional hazards models.

**Supplemental Table S1. Primary and Secondary Outcomes in the Study Population with Diabetic KTR among SGLT-2i Users (n = 1,970) Versus Non-Users (n = 1,970)**

| Outcome | Patients with outcome | | | | aHR | *p*-value* | *E*-value | |
|---|---|---|---|---|---|---|---|---|
| | 6 months | 1 year | 3 years | 5 years | (95% CI) | | For aHR | For Upper Bound of 95% CI |
| **All-cause mortality** | | | | | | | | |
| SGLT-2i users | 10 | 16 | 32 | 41 | 0.32 | <0.001 | 5.70 | 3.87 |
| SGLT-2i non-users | 18 | 45 | 136 | 188 | [0.22 - 0.45] | | | |
| **MACE†** | | | | | | | | |
| SGLT-2i users | 10 | 21 | 52 | 72 | 0.48 | <0.001 | 3.59 | 2.61 |
| SGLT-2i non-users | 22 | 62 | 168 | 229 | [0.37 - 0.62] | | | |
| **MAKE** | | | | | | | | |
| SGLT-2i users | 67 | 99 | 156 | 176 | 0.52 | <0.001 | 3.26 | 2.61 |
| SGLT-2i non-users | 111 | 199 | 353 | 444 | [0.43 - 0.62] | | | |

* The log-rank test was used to assess differences in outcome probabilities between cohorts, with significance determined at a two-sided *p*-value < 0.05.

† Patients who had experienced a MACE prior to the specified time window were excluded. After exclusion, there were 1,623 SGLT-2i users and 1,651 SGLT-2i non-users.

**Abbreviation:**

aHR, adjusted hazard ratio; CI, confidence interval; KTR, kidney transplant recipient; MACE, major adverse cardiovascular event; MAKE, major adverse kidney event; SGLT-2i, sodium-glucose cotransporter 2 inhibitor.

**Supplemental Table S2. Incidence Rate Ratios for Outcomes of Interest among SGLT-2i Users Versus Non-Users**

| Outcome | Participants, n | Patients with Outcome, n | Mean follow-up years | Crude Incidence Rate/1,000 Person-Years | Incidence Rate Ratio (95% CI) |
|---|---|---|---|---|---|
| **All-cause mortality** | 3940 | 229 | 3.0 | 19.18 | 0.20 [0.14 - 0.28] |
| SGLT-2i users | 1970 | 41 | 3.2 | 6.56 | |
| SGLT-2i non-users | 1970 | 188 | 2.9 | 33.05 | |
| **MACE*** | 3274 | 301 | 3.1 | 30.05 | 0.30 [0.23 - 0.40] |
| SGLT-2i users | 1623 | 72 | 3.1 | 14.11 | |
| SGLT-2i non-users | 1651 | 229 | 3.0 | 46.63 | |
| **MAKE** | 3940 | 620 | 3.0 | 51.93 | 0.36 [0.30 - 0.43] |
| SGLT-2i users | 1970 | 176 | 3.2 | 28.15 | |
| SGLT-2i non-users | 1970 | 444 | 2.9 | 78.06 | |

* Patients who had experienced a MACE prior to the specified time window were excluded.

**Abbreviation:**

CI, confidence interval; MACE, major adverse cardiovascular event; MAKE, major adverse kidney event; SGLT-2i, sodium-glucose cotransporter 2 inhibitor.

**Supplemental Table S3. Absolute Risks, Risk Differences, Risk Ratio, and Odds Ratio for All-Cause Mortality among SGLT-2i Users Versus Non-Users**

| | Patients with Outcome, n | Absolute Risks | Risk Difference (95% CI) | *p*-value* for Risk Difference | Risk Ratio (95% CI) | Odds Ratio (95% CI) |
|---|---|---|---|---|---|---|
| **1 year post-transplant** | | | | | | |
| SGLT-2i users | 16/1970 | 0.81% | -1.47% | <0.001 | 0.36 | 0.35 |
| SGLT-2i non-users | 45/1970 | 2.28% | [-2.24% to -0.70%] | | [0.20 - 0.63] | [0.20 - 0.62] |
| **3 years post-transplant** | | | | | | |
| SGLT-2i users | 32/1970 | 1.62% | -5.28% | <0.001 | 0.24 | 0.22 |
| SGLT-2i non-users | 136/1970 | 6.90% | [-6.53% to -4.03%] | | [0.16 - 0.34] | [0.15 - 0.33] |
| **5 years post-transplant** | | | | | | |
| SGLT-2i users | 41/1970 | 2.08% | -7.46% | <0.001 | 0.22 | 0.20 |
| SGLT-2i non-users | 188/1970 | 9.54% | [-8.90% to -6.02%] | | [0.16 - 0.30] | [0.14 - 0.28] |

* The log-rank test was used to assess differences in outcome probabilities between cohorts, with significance determined at a two-sided *p*-value < 0.05.

**Abbreviation:**

CI, confidence interval; SGLT-2i, sodium-glucose cotransporter 2 inhibitor.

**Supplemental Table S4. Outcomes Excluding Patients with Different Dialysis-Related Codes within the First 1-3 Months Post-Transplant***

| Outcome | aHR after matching (95% CI) | *p*-value† |
|---|---|---|
| **Only excluded patients with mortality or dialysis-related diagnosis codes: 2,063 SGLT-2i users vs. 28,057 non-users** | | |
| All-cause mortality | 0.35 [0.25 - 0.49] | <0.001 |
| MACE | 0.50 [0.39 - 0.63] | <0.001 |
| MAKE | 0.52 [0.45 - 0.61] | <0.001 |
| **Only excluded patients with mortality or dialysis-related procedure codes: 2,723 SGLT-2i users vs. 35,362 non-users** | | |
| All-cause mortality | 0.31 [0.23 - 0.42] | <0.001 |
| MACE | 0.53 [0.43 - 0.65] | <0.001 |
| MAKE | 0.76 [0.69 - 0.84] | <0.001 |

* In the SGLT-2i user group, 972 patients were excluded due to having dialysis-related diagnosis codes within 1-3 months post-transplantation, and 312 patients were excluded due to having dialysis-related procedure codes in the same timeframe. Among these, 244 patients were excluded for having both types of codes. In the SGLT-2i non-user group, 12,232 patients were excluded due to dialysis-related diagnosis codes, and 4,927 due to dialysis-related procedure codes within the first 1-3 months post-transplant. Of these, 3,704 patients were excluded for having both diagnostic and procedure codes. The specific codes used for these exclusions are detailed in **Supplemental Table S8**.

† The log-rank test was used to assess differences in outcome probabilities between cohorts, with significance determined at a two-sided $p$-value < 0.05.

**Abbreviation:**

aHR, adjusted hazard ratio; CI, confidence interval; MACE, major adverse cardiovascular event; MAKE, major adverse kidney event; SGLT-2i, sodium-glucose cotransporter 2 inhibitor.

**Supplemental Table S5. Comparative Analysis of Outcomes Based on SGLT-2i Usage in the First 3 Months and Continued Usage from 3 to 6 Months Post-Transplant***

| **Outcome** | **aHR after matching (95% CI)** | ***p*-value†** |
|---|---|---|
| All-cause mortality | 0.47 [0.30 - 0.73] | <0.001 |
| MACE | 0.66 [0.49 - 0.90] | 0.008 |
| MAKE | 0.56 [0.45 - 0.70] | <0.001 |

* Comparison of SGLT-2i users in the first 3 months with continued usage from 3 to 6 months post-transplant and non-users in the first 3 months with continued non-usage from 3 to 6 months post-transplant. Patients were categorized based on the presence of medication codes during these time periods to determine continued usage or non-usage of SGLT-2i.

† The log-rank test was used to assess differences in outcome probabilities between cohorts, with significance determined at a two-sided $p$-value < 0.05.

**Abbreviation:**

aHR, adjusted hazard ratio; CI, confidence interval; MACE, major adverse cardiovascular event; MAKE, major adverse kidney event; SGLT-2i, sodium-glucose cotransporter 2 inhibitor.

**Supplemental Table S6. Comparison of HbA1c Levels Between SGLT-2i Users and Non-Users at 3-6, 6-9, and 9-12 Months Post-Transplant**

| | Before matching | | | After matching | | |
|---|---|---|---|---|---|---|
| | **SGLT-2i users** | **SGLT-2i non-users** | ***p*-value*** | **SGLT-2i users** | **SGLT-2i non-users** | ***p*-value*** |
| **Post-transplant HbA1c (%)†** | | | | | | |
| 3 to 6 months | 7.5 ± 1.6 | 7.1 ± 1.7 | <0.001 | 7.5 ± 1.6 | 7.4 ± 1.6 | 0.049 |
| 6 to 9 months | 7.5 ± 1.5 | 7.1 ± 1.7 | <0.001 | 7.5 ± 1.5 | 7.4 ± 1.7 | 0.262 |
| 9 to 12 months | 7.5 ± 1.5 | 7.2 ± 1.7 | <0.001 | 7.5 ± 1.5 | 7.4 ± 1.7 | 0.393 |

* The log-rank test was used to assess differences in outcome probabilities between cohorts, with significance determined at a two-sided $p$-value < 0.05.

† Plus–minus values are means ±SD.

**Abbreviation:**

HbA1c, hemoglobin A1c; SGLT-2i, sodium-glucose cotransporter 2 inhibitor.

**Supplemental Table S7. Code Use for Query Criteria of Diabetic Kidney Transplant Recipients (KTR)**

| Code use for Kidney Transplant | | | |
|---|---|---|---|
| **The cohort** | **Code type** | **Code** | **Code name** |
| Patients must have any of | diagnosis | UMLS:ICD10CM:Z94.0 | Kidney transplant status |
| | procedure | UMLS:ICD10PCS:0TY00Z0 | Transplantation of Right Kidney, Allogeneic, Open Approach |
| | procedure | UMLS:ICD10PCS:0TY10Z0 | Transplantation of Left Kidney, Allogeneic, Open Approach |
| | procedure | UMLS:ICD10PCS:0TY10Z1 | Transplantation of Left Kidney, Syngeneic, Open Approach |
| | procedure | UMLS:ICD10PCS:0TY00Z1 | Transplantation of Right Kidney, Syngeneic, Open Approach |
| | procedure | UMLS:ICD10PCS:0TY10Z2 | Transplantation of Left Kidney, Zooplastic, Open Approach |
| | procedure | UMLS:ICD10PCS:0TY00Z2 | Transplantation of Right Kidney, Zooplastic, Open Approach |
| | procedure | UMLS:SNOMED:70536003 | Transplant of kidney |
| | procedure | UMLS:SNOMED:175902000 | Cadaveric renal transplant |
| | procedure | UMLS:CPT:1008098 | Renal Transplantation Procedures |
| **Date constraint** | The terms in this group occurred between Jun 1, 2015 and Jun 1, 2023 | | |

| Code use for Type 2 Diabetes | | | |
|---|---|---|---|
| **The cohort** | **Code type** | **Code** | **Code name** |
| Patients must have | diagnosis | UMLS:ICD10CM:E11 | Type 2 diabetes mellitus |

**Supplemental Table S8. Code Use for Query Criteria of Sodium-Glucose Co-Transporter 2 Inhibitor (SGLT-2i) and Exclusion**

| Code use for SGLT-2i | | | | |
|---|---|---|---|---|
| **The cohort** | | **Code type** | **Code** | **Code name** |
| Patients must have (SGLT-2i users)<br>Patients cannot have (SGLT-2i non-users) | | medication | NLM:ATC:A10BK | Sodium-glucose co-transporter 2 (SGLT2) inhibitors |

| Code use for Exclusion | | | | |
|---|---|---|---|---|
| **The cohort** | | **Code type** | **Code** | **Code name** |
| Patients cannot have | | demographics | Deceased | Deceased |
| | or | diagnosis | UMLS:ICD10CM:R99 | Ill-defined and unknown cause of mortality |
| | or | procedure | UMLS:CPT:90937 | Hemodialysis procedure requiring repeated evaluation(s) with or without substantial revision of dialysis prescription |
| | or | procedure | UMLS:CPT:90947 | Dialysis procedure other than hemodialysis (eg, peritoneal dialysis, hemofiltration, or other continuous renal replacement therapies) requiring repeated evaluations by a physician or other qualified health care professional, with or without substantial revision of dialysis prescription |
| | or | procedure | UMLS:CPT:90945 | Dialysis procedure other than hemodialysis (eg, peritoneal dialysis, hemofiltration, or other continuous renal replacement therapies), with single evaluation by a physician or other qualified health care professional |
| | or | procedure | UMLS:CPT:1012752 | Hemodialysis Procedures |
| | or | procedure | UMLS:CPT:90935 | Hemodialysis procedure with single evaluation by a physician or other qualified health care professional |
| | or | procedure | UMLS:SNOMED:302497006 | Hemodialysis |

| | | | |
|---|---|---|---|
| or | procedure | UMLS:CPT:1012740 | Dialysis Services and Procedures |
| or | diagnosis | UMLS:ICD10CM:Z99.2 | Dependence on renal dialysis |

**Supplemental Table S9. Compare Outcome Definitions**

| | | | |
|---|---|---|---|
| **Mortality** | | | |
| | **Code type** | **Code** | **Code name** |
| | Demographics | Deceased | Deceased |
| | Diagnosis | UMLS:ICD10CM:R99 | Ill-defined and unknown cause of mortality |
| **Settings for the performed analyses** | | | |
| | Kaplan - Meier survival analysis | | excluding patients with outcome prior to the time window |
| **Major adverse cardiovascular event (MACE)** | | | |
| | **Code type** | **Code** | **Code name** |
| | Demographics | Deceased | Deceased |
| | Diagnosis | UMLS:ICD10CM:R99 | Ill-defined and unknown cause of mortality |
| | Diagnosis | UMLS:ICD10CM:I21 | Acute myocardial infarction |
| | Diagnosis | UMLS:ICD10CM:I46 | Cardiac arrest |
| | Diagnosis | UMLS:ICD10CM:I61 | Nontraumatic intracerebral hemorrhage |
| | Diagnosis | UMLS:ICD10CM:I63 | Cerebral infarction |
| **Settings for the performed analyses** | | | |
| | Kaplan - Meier survival analysis | | excluding patients with outcome prior to the time window |
| **Major adverse kidney event (MAKE)** | | | |
| | **Code type** | **Code** | **Code name** |
| | Demographics | NA | Deceased |
| | Diagnosis | UMLS:ICD10CM:R99 | Ill-defined and unknown cause of mortality |
| | Procedure | UMLS:CPT:90937 | Hemodialysis procedure requiring repeated evaluation(s) with or without substantial revision of dialysis prescription |
| | Procedure | UMLS:CPT:90947 | Dialysis procedure other than hemodialysis (eg, peritoneal dialysis, hemofiltration, or other continuous renal replacement therapies) requiring repeated evaluations by a physician or other qualified health care professional, with or without substantial revision of dialysis prescription |
| | Procedure | UMLS:CPT:90945 | Dialysis procedure other than hemodialysis (eg, peritoneal dialysis, hemofiltration, or other continuous renal replacement therapies), with single evaluation by a physician or other qualified health care professional |
| | Procedure | UMLS:CPT:1012752 | Hemodialysis Procedures |
| | Procedure | UMLS:CPT:90935 | Hemodialysis procedure with single evaluation by a physician or other qualified health care professional |
| | Procedure | UMLS:SNOMED:302497006 | Hemodialysis |
| | Procedure | UMLS:CPT:1012740 | Dialysis Services and Procedures |

| | | |
|---|---|---|
| Diagnosis | UMLS:ICD10CM:Z99.2 | Dependence on renal dialysis |
| Laboratory | TNX:8001 | Glomerular filtration rate/1.73 sq M.predicted [Volume Rate/Area] in Serum, Plasma or Blood by Creatinine-based formula (MDRD) (at most 14.00 mL/min/{1.73_m2} (most recent occurrence)) |
| **Settings for the performed analyses** | | |
| Kaplan - Meier survival analysis | | including patients with outcome prior to the time window |
| **Diabetic ketoacidosis (DKA)** | | |
| **Code type** | **Code** | **Code name** |
| Diagnosis | UMLS:ICD10CM:E11.1 | Type 2 diabetes mellitus with ketoacidosis |
| **Settings for the performed analyses** | | |
| Kaplan - Meier survival analysis | | excluding patients with outcome prior to the time window |
| **Osteoporotic fracture** | | |
| **Code type** | **Code** | **Code name** |
| Diagnosis | UMLS:ICD10CM:M80 | Osteoporosis with current pathological fracture |
| **Settings for the performed analyses** | | |
| Kaplan - Meier survival analysis | | excluding patients with outcome prior to the time window |
| **Appendicitis** | | |
| **Code type** | **Code** | **Code name** |
| Diagnosis | UMLS:ICD10CM:K35 | Acute appendicitis |
| Diagnosis | UMLS:ICD10CM:K36 | Other appendicitis |
| Diagnosis | UMLS:ICD10CM:K37 | Unspecified appendicitis |
| **Settings for the performed analyses** | | |
| Kaplan - Meier survival analysis | | excluding patients with outcome prior to the time window |
| **Hematologic malignancy** | | |
| **Code type** | **Code** | **Code name** |
| Diagnosis | UMLS:ICD10CM:C81-C96 | Malignant neoplasms of lymphoid, hematopoietic and related tissue |
| **Settings for the performed analyses** | | |
| Kaplan - Meier survival analysis | | excluding patients with outcome prior to the time window |
| **Common cold** | | |
| **Code type** | **Code** | **Code name** |
| Diagnosis | UMLS:ICD10CM:J00 | Acute nasopharyngitis [common cold] |
| **Settings for the performed analyses** | | |
| Kaplan - Meier survival analysis | | excluding patients with outcome prior to the time window |
| **Irritable bowel syndrome** | | |
| **Code type** | **Code** | **Code name** |
| Diagnosis | UMLS:ICD10CM:K58 | Irritable bowel syndrome |
| **Settings for the performed analyses** | | |

| | | |
|---|---|---|
| Kaplan - Meier survival analysis | | excluding patients with outcome prior to the time window |
| **Burn** | | |
| **Code type** | **Code** | **Code name** |
| Diagnosis | UMLS:ICD10CM:T20-T25 | Burns and corrosions of external body surface, specified by site |
| **Settings for the performed analyses** | | |
| Kaplan - Meier survival analysis | | excluding patients with outcome prior to the time window |
| **Suicide attempt & ideation** | | |
| **Code type** | **Code** | **Code name** |
| Diagnosis | UMLS:ICD10CM:T14.91 | Suicide attempt |
| Diagnosis | UMLS:ICD10CM:R45.851 | Suicidal ideations |
| **Settings for the performed analyses** | | |
| Kaplan - Meier survival analysis | | excluding patients with outcome prior to the time window |
| **Acute myocardial infarction (AMI)** | | |
| **Code type** | **Code** | **Code name** |
| Diagnosis | UMLS:ICD10CM:I21 | Acute myocardial infarction |
| **Settings for the performed analyses** | | |
| Kaplan - Meier survival analysis | | excluding patients with outcome prior to the time window |
| **Stroke** | | |
| **Code type** | **Code** | **Code name** |
| Diagnosis | UMLS:ICD10CM:I61 | Nontraumatic intracerebral hemorrhage |
| Diagnosis | UMLS:ICD10CM:I63 | Cerebral infarction |
| **Settings for the performed analyses** | | |
| Kaplan - Meier survival analysis | | excluding patients with outcome prior to the time window |
| **Incident dialysis** | | |
| **Code type** | **Code** | **Code name** |
| Procedure | UMLS:CPT:90937 | Hemodialysis procedure requiring repeated evaluation(s) with or without substantial revision of dialysis prescription |
| Procedure | UMLS:CPT:90947 | Dialysis procedure other than hemodialysis (eg, peritoneal dialysis, hemofiltration, or other continuous renal replacement therapies) requiring repeated evaluations by a physician or other qualified health care professional, with or without substantial revision of dialysis prescription |
| Procedure | UMLS:CPT:90945 | Dialysis procedure other than hemodialysis (eg, peritoneal dialysis, hemofiltration, or other continuous renal replacement therapies), with single evaluation by a physician or other qualified health care professional |

| | | |
|---|---|---|
| Procedure | UMLS:CPT:90935 | Hemodialysis procedure with single evaluation by a physician or other qualified health care professional |
| Procedure | UMLS:CPT:1012752 | Hemodialysis Procedures |
| Procedure | UMLS:SNOMED:302497006 | Hemodialysis |
| Procedure | UMLS:CPT:1012740 | Dialysis Services and Procedures |
| Diagnosis | UMLS:ICD10CM:Z99.2 | Dependence on renal dialysis |
| **Settings for the performed analyses** | | |
| Kaplan - Meier survival analysis | | including patients with outcome prior to the time window |
| **Acute kidney injury (AKI)** | | |
| **Code type** | **Code** | **Code name** |
| Diagnosis | UMLS:ICD10CM:N17 | Acute kidney failure |
| **Settings for the performed analyses** | | |
| Kaplan - Meier survival analysis | | including patients with outcome prior to the time window |
| **Urinary tract infection (UTI)** | | |
| **Code type** | **Code** | **Code name** |
| Diagnosis | UMLS:ICD10CM:N39.0 | Urinary tract infection, site not specified |
| Diagnosis | UMLS:ICD10CM:N30 | Cystitis |
| Diagnosis | UMLS:ICD10CM:N10 | Acute pyelonephritis |
| **Settings for the performed analyses** | | |
| Kaplan - Meier survival analysis | | excluding patients with outcome prior to the time window |
| **Urogenital candidiasis** | | |
| **Code type** | **Code** | **Code name** |
| Diagnosis | UMLS:ICD10CM:B37.3 | Candidiasis of vulva and vagina |
| Diagnosis | UMLS:ICD10CM:B37.4 | Candidiasis of other urogenital sites |
| **Settings for the performed analyses** | | |
| Kaplan - Meier survival analysis | | excluding patients with outcome prior to the time window |

**Supplemental Table S10. Diagnosis and Medication Codes Used in the Study**

| **Diagnosis Code** | | | |
|---|---|---|---|
| | **Disease** | **Code (ICD-10-CM)** | **Code name** |
| | Hypertension | I10 | Essential (primary) hypertension |
| | Heart failure | I50 | Heart failure |
| | DM nephropathy | E11.2 | Type 2 diabetes mellitus with kidney complications |
| | DM ophthalmology | E11.3 | Type 2 diabetes mellitus with ophthalmic complications |
| | DM neuropathy | E11.4 | Type 2 diabetes mellitus with neurological complications |
| | Liver diseases | K70-K77 | Diseases of liver |
| | Chronic lower respiratory diseases | J40-J4A | Chronic lower respiratory diseases |
| | Systemic connective tissue disorders | M30-M36 | Systemic connective tissue disorders |
| | Neoplasms | C00-D49 | Neoplasms |
| | Dyslipidemia | E78 | Disorders of lipoprotein metabolism and other lipidemias |
| | Obesity | E66 | Overweight and obesity |
| | Nephrotic syndrome | N04 | Nephrotic syndrome |
| | Cystic kidney disease | Q61 | Cystic kidney disease |
| | Gout | M10 | Gout |
| | Proteinuria | R80 | Proteinuria |
| | Smoking | Z72.0 | Tobacco use |
| **Medication Code** | | | |
| | **Drug** | **Code** | **Code name** |
| | Corticosteroids | ATC:H02 | CORTICOSTEROIDS FOR SYSTEMIC USE |
| | Insulin | ATC:A10A | INSULINS AND ANALOGUES |
| | Biguanides | ATC:A10BA | Biguanides |
| | Glucagon-like peptide-1 (GLP-1) agonists | ATC:A10BJ | Glucagon-like peptide-1 (GLP-1) analogues |
| | Dipeptidyl peptidase 4 (DPP-4) inhibitors | ATC:A10BH | Dipeptidyl peptidase 4 (DPP-4) inhibitors |
| | Sulfonylureas | ATC:A10BB | Sulfonylureas |
| | Thiazolidinediones | ATC:A10BG | Thiazolidinediones |
| | HMG-CoA reductase inhibitors | ATC:C10AA | HMG CoA reductase inhibitors |
| | Angiotensin-converting enzyme | ATC:C09 | AGENTS ACTING ON THE RENIN-ANGIOTENSIN SYSTEM |

| | | |
|---|---|---|
| inhibitor/Angiotensin II receptor blocker | | |
| Beta-blockers | ATC:C07 | BETA BLOCKING AGENTS |
| Calcium channel blocker | ATC:C08 | CALCIUM CHANNEL BLOCKERS |
| Diuretics | ATC:C03 | DIURETICS |
| Vitamin C | ATC:A11G | ASCORBIC ACID (VITAMIN C), INCL. COMBINATIONS |
| Tacrolimus | RXNORM:42316 | tacrolimus |
| Mycophenolate mofetil (MMF) | RXNORM:68149 | mycophenolate mofetil |

**Supplemental Figure S1. Kaplan-Meier Survival Curves for Outcomes of Interest.** (A) All-cause mortality; (B) Major adverse cardiovascular events (MACE); (C) Major adverse kidney events (MAKE). Source data are provided as a Source Data file.

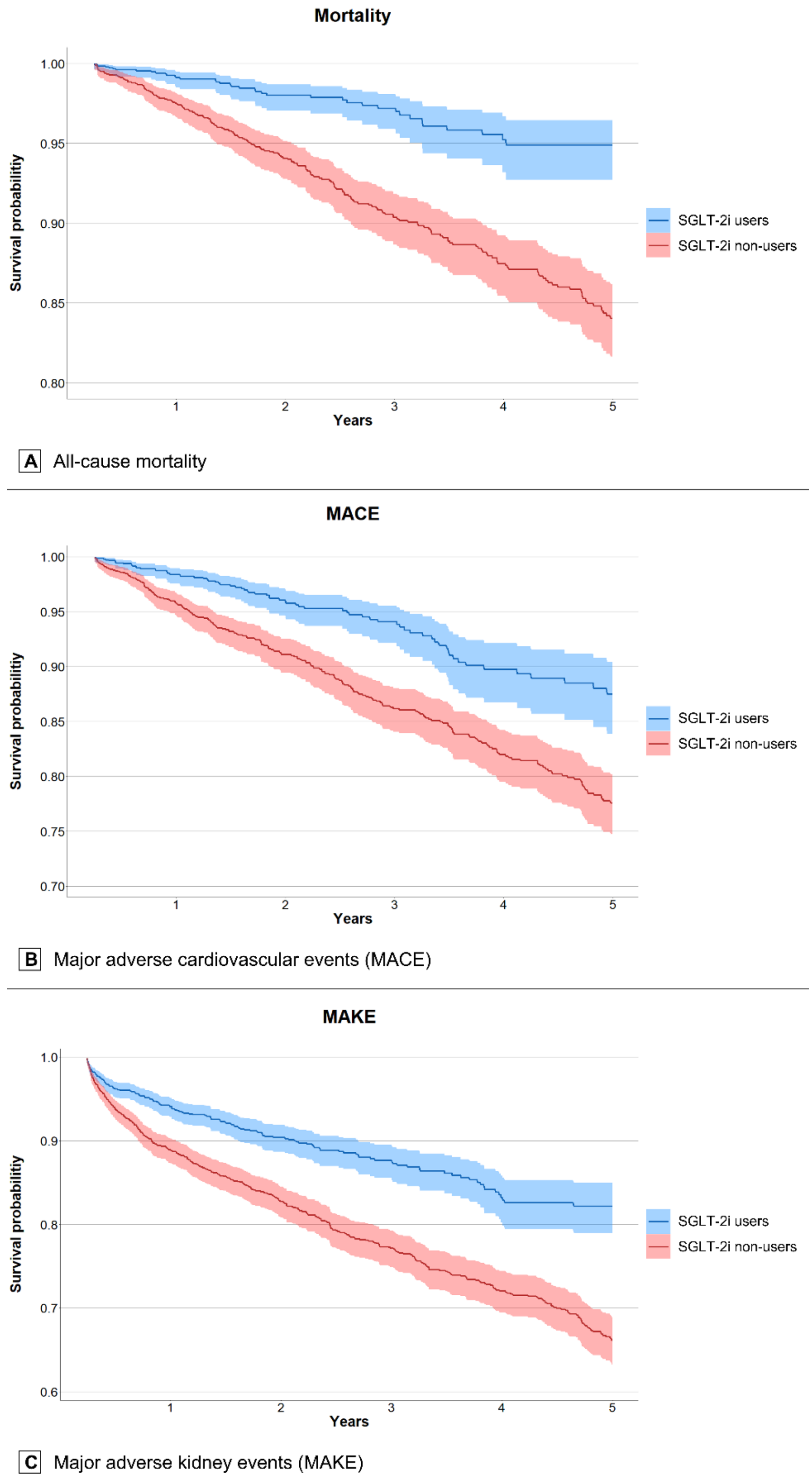


**Abbreviation:**

MACE, major adverse cardiovascular event; MAKE, major adverse kidney event; SGLT-2i, sodium-glucose cotransporter 2 inhibitor.

**Supplemental Figure S2. Graphical Abstract**

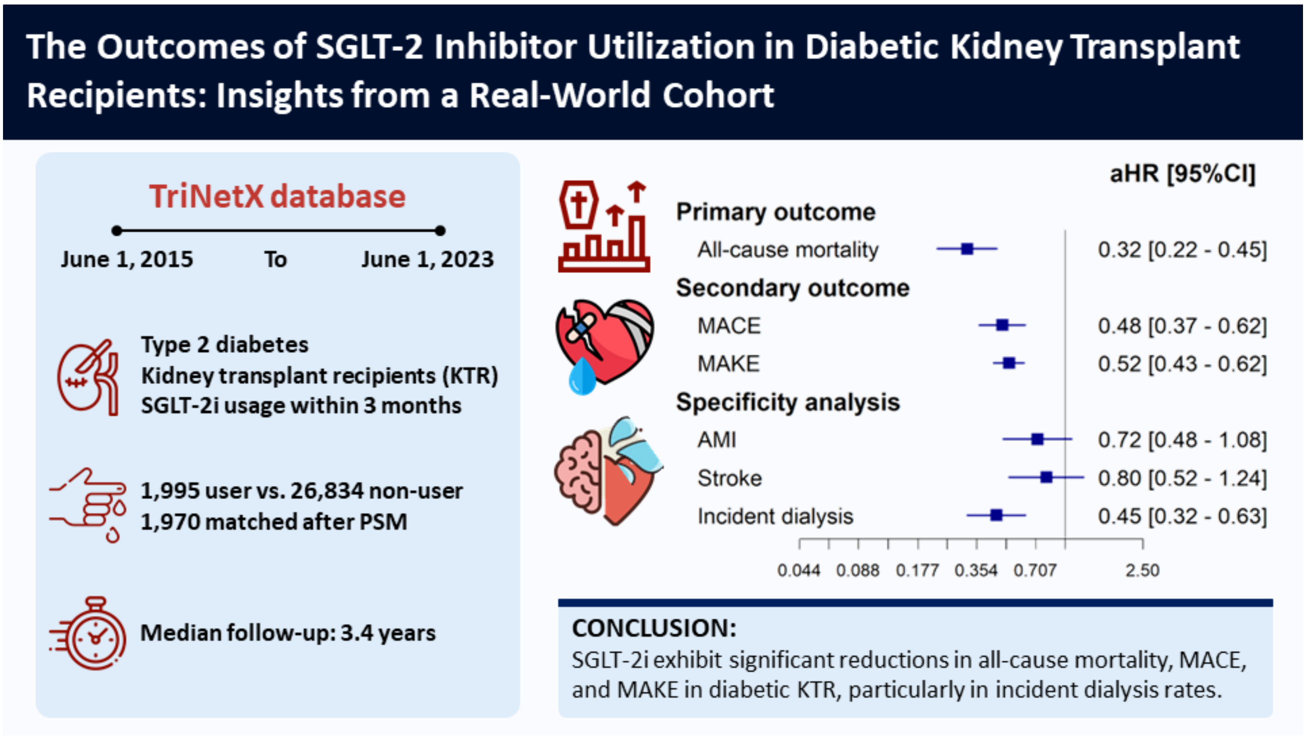


**Abbreviation:**

aHR, adjusted hazard ratio; AMI, acute myocardial infarction; CI, confidence interval; KTR, kidney transplant recipient; MACE, major adverse cardiovascular event; MAKE, major adverse kidney event; PSM, propensity score matching; SGLT-2i, sodium-glucose cotransporter 2 inhibitor.
