# Supplementary material for: The outcomes of SGLT-2 inhibitor utilization in diabetic kidney transplant recipients
Source: Nat Commun. 2024 Nov 20;15:10043. doi: 10.1038/s41467-024-54171-8 (PMC11579355; doi:10.1038/s41467-024-54171-8)
Supplement: Supplementary file 2 — Reporting Summary [file 41467_2024_54171_MOESM2_ESM.pdf]

nature portfolio

Corresponding author(s): Jeff S. Chueh, Vin-Cent Wu

Last updated by author(s): Oct 5, 2024

Reporting Summary

Nature Portfolio wishes to improve the reproducibility of the work that we publish. This form provides structure for consistency and transparency in reporting. For further information on Nature Portfolio policies, see our Editorial Policies and the Editorial Policy Checklist.

Statistics

For all statistical analyses, confirm that the following items are present in the figure legend, table legend, main text, or Methods section.

n/a	Confirmed	
☐	☒	The exact sample size (n) for each experimental group/condition, given as a discrete number and unit of measurement
☐	☒	A statement on whether measurements were taken from distinct samples or whether the same sample was measured repeatedly
☐	☒	The statistical test(s) used AND whether they are one- or two-sided<br>*Only common tests should be described solely by name; describe more complex techniques in the Methods section.*
☐	☒	A description of all covariates tested
☐	☒	A description of any assumptions or corrections, such as tests of normality and adjustment for multiple comparisons
☐	☒	A full description of the statistical parameters including central tendency (e.g. means) or other basic estimates (e.g. regression coefficient) AND variation (e.g. standard deviation) or associated estimates of uncertainty (e.g. confidence intervals)
☐	☒	For null hypothesis testing, the test statistic (e.g. F, t, r) with confidence intervals, effect sizes, degrees of freedom and P value noted<br>*Give P values as exact values whenever suitable.*
☒	☐	For Bayesian analysis, information on the choice of priors and Markov chain Monte Carlo settings
☒	☐	For hierarchical and complex designs, identification of the appropriate level for tests and full reporting of outcomes
☒	☐	Estimates of effect sizes (e.g. Cohen's d, Pearson's r), indicating how they were calculated

Our web collection on statistics for biologists contains articles on many of the points above.

Software and code

Policy information about availability of computer code

Data collection	This research utilized a historical cohort dataset obtained from the TriNetX network, a significant collection predominantly made up of electronic health record data, enriched with additional laboratory and mortality information. The dataset extends up to the present and includes data on over 100 million individuals. It provides a diverse range of data, including patient demographics, medical diagnoses (coded using ICD-10-CM), various medical procedures (recorded with ICD-10 Procedure Coding System, Current Procedural Terminology codes, or Systematized Nomenclature of Medicine - Clinical Terms), prescribed drugs (categorized by the Anatomical Therapeutic Chemical Classification system or RxNorm), laboratory tests (classified by Logical Observation Identifiers Names and Codes), genetic data (notated according to the Human Genome Variation Society standards), and detailed records of healthcare service use. This dataset was securely accessed for this study in January 2024 and includes data collected from June 1, 2015, to June 1, 2023, within the TriNetX Network.
Data analysis	Basic statistical analyses in this study were conducted using the built-in statistical system of the TriNetX platform. Additional analyses were performed using R software (version 3.2.2, Free Software Foundation, Boston, MA).

For manuscripts utilizing custom algorithms or software that are central to the research but not yet described in published literature, software must be made available to editors and reviewers. We strongly encourage code deposition in a community repository (e.g. GitHub). See the Nature Portfolio guidelines for submitting code & software for further information.

Data

Policy information about availability of data

All manuscripts must include a data availability statement. This statement should provide the following information, where applicable:

- Accession codes, unique identifiers, or web links for publicly available datasets
- A description of any restrictions on data availability
- For clinical datasets or third party data, please ensure that the statement adheres to our policy

The aggregated datasets generated and analyzed during this study were obtained from the TriNetX platform. We do not have access to individual-level data due to the TriNetX's data sharing policies. Source data are provided with this paper.

Research involving human participants, their data, or biological material

Policy information about studies with human participants or human data. See also policy information about sex, gender (identity/presentation), and sexual orientation and race, ethnicity and racism.

Reporting on sex and gender	In our study, we have reported exclusively on 'sex' as we investigated biological factors. The necessary distribution of study participants or samples has been presented in the 'Results' section of our report.
Reporting on race, ethnicity, or other socially relevant groupings	In this study, the racial and ethnic categories represented were 'White' and 'Not Hispanic or Latino'. These data were provided by healthcare organizations (HCOs) through the TriNetX database. We included this demographic information in our analysis to explore potential variations in the effects of sodium-glucose cotransporter 2 inhibitors (SGLT-2i) across these groups, thereby enhancing the robustness and generalizability of our findings.
Population characteristics	In the cohort consisting of 28,829 diabetic kidney transplant recipients (KTRs), we refined our analysis by identifying 1,995 diabetic KTRs with SGLT-2i users who neither required dialysis nor passed away within 1 to 3 months. A comparable group of 26,834 non-users was also selected. The SGLT-2i users were older than the SGLT-2i non-users (mean age 59.5 ± 11.3 years vs 57.2 ± 12.3 years). There was a higher proportion of males in the SGLT-2i group compared to the SGLT-2i non-users (63.4% vs 58.6%). The proportion of individuals of White ethnicity was 36.0% in the SGLT-2i user group, compared to 49.9% in the non-user group.
Recruitment	Our inclusion criteria encompassed patients with type 2 diabetes, aged over 18 years, who were administered SGLT-2i within the 3 months post-transplant.
Ethics oversight	This study complies with all relevant ethical regulations and was approved by the Institutional Review Board (IRB) of Chi-Mei Medical Center, Tainan, Taiwan (Approval Numbers: 11202-002, 11210-E01).

Note that full information on the approval of the study protocol must also be provided in the manuscript.

Field-specific reporting

Please select the one below that is the best fit for your research. If you are not sure, read the appropriate sections before making your selection.

☒ Life sciences ☐ Behavioural & social sciences ☐ Ecological, evolutionary & environmental sciences

For a reference copy of the document with all sections, see nature.com/documents/nr-reporting-summary-flat.pdf

Life sciences study design

All studies must disclose on these points even when the disclosure is negative.

Sample size	In this study, the sample size was determined based on the statistical power required to detect significant effects of SGLT-2 inhibitors on cardio-renal outcomes and mortality in diabetic kidney transplant recipients. We performed sample size calculations using standard power analysis techniques, targeting a power of 90% and an alpha of 0.05 to ensure sufficient sensitivity for detecting clinically relevant differences. Preliminary estimates of effect sizes were based on prior literature of similar interventions. This rationale allowed us to ensure that the chosen sample sizes were sufficient for robust and valid conclusions.
Data exclusions	The patients who died or required dialysis between 1 to 3 months post-transplant were excluded.
Replication	We have utilized landmark analysis to assess whether the observed effects remain consistent across different timeframes and have confirmed the replication of our findings to ensure the robustness and reliability of our results.
Randomization	This is a retrospective observation cohort study, so we did not have any design about randomization.
Blinding	This is a retrospective observation cohort study, so we did not have any design about blinding.

Reporting for specific materials, systems and methods

We require information from authors about some types of materials, experimental systems and methods used in many studies. Here, indicate whether each material, system or method listed is relevant to your study. If you are not sure if a list item applies to your research, read the appropriate section before selecting a response.

Materials & experimental systems

n/a	Involved in the study
☒	☐ Antibodies
☒	☐ Eukaryotic cell lines
☒	☐ Palaeontology and archaeology
☒	☐ Animals and other organisms
☐	☒ Clinical data
☒	☐ Dual use research of concern
☒	☐ Plants

Methods

n/a	Involved in the study
☒	☐ ChIP-seq
☒	☐ Flow cytometry
☒	☐ MRI-based neuroimaging

Clinical data

Policy information about clinical studies

All manuscripts should comply with the ICMJEguidelines for publication of clinical research and a completedCONSORT checklist must be included with all submissions.

Clinical trial registration	This is a retrospective observation cohort study, so we do not have registration number.
Study protocol	In our research, we focused on KTR diagnosed with type 2 diabetes, as classified under the ICD-10-CM code E11. The index date for these patients was defined as the date of their kidney transplant, determined using the ICD-10-CM code Z94.0, along with associated procedure codes for kidney transplantation. Our inclusion criteria encompassed patients with type 2 diabetes, aged over 18 years, who were administered SGLT-2i within the 3 months post-transplant. The study's control group comprised diabetic KTR who did not receive SGLT-2i within the 3 months post-transplant. We deliberately excluded patients in both the user and non-user groups who died or required dialysis between 1 to 3 months post-transplant, as dialysis dependency during this period may indicate graft failure, alongside the restriction on the use of SGLT-2i in patients undergoing dialysis. This exclusion aligns with the landmark method, minimizing the possibility of an immortal time interval. To achieve an equitable distribution of covariates across the primary study group and a comparison group, our approach involved using TriNetX's built-in statistics to match the two groups in a 1:1 propensity score matching (PSM). This method was applied to a comprehensive set of 45 characteristics, covering a wide range of categories, including patient demographics (such as age and sex), medical history (e.g., previous diagnoses), concomitant medications, and laboratory test results.
Data collection	This research utilized a historical cohort dataset obtained from the TriNetX network, a significant collection predominantly made up of electronic health record data, enriched with additional laboratory and mortality information. The dataset extends up to the present and includes data on over 100 million individuals. It provides a diverse range of data, including patient demographics, medical diagnoses (coded using ICD-10-CM), various medical procedures (recorded with ICD-10 Procedure Coding System, Current Procedural Terminology codes, or Systematized Nomenclature of Medicine - Clinical Terms), prescribed drugs (categorized by the Anatomical Therapeutic Chemical Classification system or RxNorm), laboratory tests (classified by Logical Observation Identifiers Names and Codes), genetic data (notated according to the Human Genome Variation Society standards), and detailed records of healthcare service use. This dataset was securely accessed for this study in January 2024 and includes data collected from June 1, 2015, to June 1, 2023, within the TriNetX Network.
Outcomes	This study primarily aimed to evaluate all-cause mortality, while also closely monitoring major adverse cardiac events (MACE), and major adverse kidney events (MAKE) as secondary outcomes. The MACE we observed included cerebral infarction, hemorrhagic stroke, acute myocardial infarction (AMI), cardiac arrest, and death. MAKE, on the other hand, encompassed events such as the initiation of re-dialysis, incident dialysis, or death. These outcomes were meticulously tracked from the 90th day post the index date and up to 5 years. To mitigate protopathic or ascertainment bias, any events of secondary outcomes except MAKE that occurred before the index date were excluded, and repeat PSM was performed.

Plants

Seed stocks	*Report on the source of all seed stocks or other plant material used. If applicable, state the seed stock centre and catalogue number. If plant specimens were collected from the field, describe the collection location, date and sampling procedures.*
Novel plant genotypes	*Describe the methods by which all novel plant genotypes were produced. This includes those generated by transgenic approaches, gene editing, chemical/radiation-based mutagenesis and hybridization. For transgenic lines, describe the transformation method, the number of independent lines analyzed and the generation upon which experiments were performed. For gene-edited lines, describe the editor used, the endogenous sequence targeted for editing, the targeting guide RNA sequence (if applicable) and how the editor was applied.*
Authentication	*Describe any authentication procedures for each seed stock used or novel genotype generated. Describe any experiments used to assess the effect of a mutation and, where applicable, how potential secondary effects (e.g. second site T-DNA insertions, mosiacism, off-target gene editing) were examined.*
